# Retinal layer thickness as biomarker for cognitive decline in rhesus macaques

**DOI:** 10.1002/alz70856_103383

**Published:** 2025-12-26

**Authors:** Yin Allison Liu

**Affiliations:** ^1^ UC Davis, Sacramento, CA, USA

## Abstract

**Background:**

Retinal layer thinning measured by optical coherence tomography (OCT) has been proposed as a noninvasive biomarker for central nervous system degeneration, including Alzheimer's disease (AD). Non‐human primates (NHPs), whose brains and retinas closely resemble those of humans, offer a promising model for preclinical testing. However, no studies have assessed the relationship between retinal layer thickness and cognitive decline in NHPs.

**Methods:**

At the California National Primate Research Center, we evaluated 110 adult rhesus macaques aged 19.54 ± 3.28 years (range 11–30; 14 males, 96 females). Biobehavioral assessments investigated included visual acuity, decision‐making, working memory, spatial memory, problem‐solving, and social learning. OCT scans of both eyes were obtained and analyzed using customized software to measure peripapillary and macular layer thicknesses within different circular regions centered on the fovea of the early treatment of diabetic retinopathy study (EDTRS) grid. Pearson correlation and multiple linear regression were used to assess relationships between retinal and optic nerve layer thickness and cognitive scores.

**Results:**

Behavioral studies revealed that older monkeys demonstrated improved decision‐making in food choice tests but worsened problem‐solving in food retrieval tasks. Visual acuity, spatial memory, and social learning behaviors remained unchanged with age. Retinal layer thickness measurements were very similar to human data and there was no age or behavior‐related changes. The behavioral data demonstrated a much larger variability compared to retinal OCT measurements.

**Conclusions:**

Rhesus macaques showed aging behaviors and retinal layer measurements similar to humans. Although behavioral testing poses challenges, retinal imaging was highly reproducible. A larger, longitudinal study pairing behavioral assessments with retinal measurements is needed to determine if the retina could serve as a noninvasive biomarker of cognitive function in preclinical NHP research.

